# Interaction of Quercetin, Cyanidin, and Their *O*-Glucosides with Planar Lipid Models: Implications for Their Biological Effects

**DOI:** 10.3390/membranes13060600

**Published:** 2023-06-14

**Authors:** Daniela Meleleo, Pinarosa Avato, Filomena Conforti, Maria Pia Argentieri, Giovanni Messina, Giuseppe Cibelli, Rosanna Mallamaci

**Affiliations:** 1 Department of Science of Agriculture, Food, Natural Resources and Engineering, University of Foggia, 71122 Foggia, Italy; 2 Department of Pharmacy-Drug Sciences, University of Bari “Aldo Moro”, 70125 Bari, Italy; pinaavato@gmail.com (P.A.); mariapia.argentieri@uniba.it (M.P.A.); 3 Department of Pharmacy, Health and Nutritional Sciences, University of Calabria, 87036 Rende, Italy; filomena.conforti@unical.it; 4 Department of Clinical and Experimental Medicine, University of Foggia, 71122 Foggia, Italy; giovanni.messina@unifg.it (G.M.); giuseppe.cibelli@unifg.it (G.C.); 5 Department of Biosciences, Biotechnologies and Environment, University of Bari “Aldo Moro”, 70125 Bari, Italy

**Keywords:** quercetin, quercetin-4′-*O*-glucoside, cyanidin, cyanidin-3-*O*-glucoside, polyphenols, antioxidant, interface active compounds, planar lipid membrane, conductive unit

## Abstract

Flavonoids are specialized metabolites produced by plants, as free aglycones or as glycosylated derivatives, which are particularly endowed with a variety of beneficial health properties. The antioxidant, anti-inflammatory, antimicrobial, anticancer, antifungal, antiviral, anti-Alzheimer’s, anti-obesity, antidiabetic, and antihypertensive effects of flavonoids are now known. These bioactive phytochemicals have been shown to act on different molecular targets in cells including the plasma membrane. Due to their polyhydroxylated structure, lipophilicity, and planar conformation, they can either bind at the bilayer interface or interact with the hydrophobic fatty acid tails of the membrane. The interaction of quercetin, cyanidin, and their *O*-glucosides with planar lipid membranes (PLMs) similar in composition to those of the intestine was monitored using an electrophysiological approach. The obtained results show that the tested flavonoids interact with PLM and form conductive units. The modality of interaction with the lipids of the bilayer and the alteration of the biophysical parameters of PLMs induced by the tested substances provided information on their location in the membrane, helping to elucidate the mechanism of action which underlies some pharmacological properties of flavonoids. To our knowledge, the interaction of quercetin, cyanidin, and their *O*-glucosides with PLM surrogates of the intestinal membrane has never been previously monitored.

## 1. Introduction

Specialized metabolites produced by plants have gained an increasing interest as health-promoting compounds. Among them, the widely distributed flavonoids are particularly endowed with a variety of beneficial health properties. Flavonoids are polyphenolic phytochemicals grouped into subclasses according to their molecular structure and the degree of oxidation of the core pyran ring. They include flavonols, flavanones, flavones, catechins, isoflavones, dihydroflavonols, chalcones, and anthocyanidins. Flavonoids can occur in plants both as free aglycones or as glycosylated derivatives. Due to their polyhydroxylated structure they have a very high antioxidant capacity, which likely can explain the numerous pharmacological properties that have been highlighted in in vivo, in vitro, and epidemiological investigations [1]. The antioxidant activity of quercetin-3-*O*-glucoside and quercetin-4′-*O*-glucoside on iron-ion-produced lipid peroxidation of rat gastrointestinal mucosa was also evaluated. The results of this study indicated that quercetin-4′-*O*-glucoside inhibits lipid peroxidation significantly more than the 3-*O*-glucoside derivative [2]. Thus, flavonoids have been proven to have anti-inflammatory, antimicrobial, anticancer, antifungal, antiviral, anti-Alzheimer’s, anti-obesity, antidiabetic, and antihypertensive effects [3,4,5].

These bioactive phytochemicals have been shown to act on different molecular targets in cells including the plasma membrane, some intracellular enzymes or receptors, and even DNA. To carry out their biological activities, these compounds must then enter the cell, cross the plasma membrane, and, therefore, interact with membrane lipids. Flavonoids are very reactive molecules which can either bind at the bilayer interface or enter the non-polar domain of the cell membrane. The different interaction of these molecules with lipids can be attributed to their lipophilicity, planar conformation, or their location/orientation within the lipid bilayer which may influence some membrane characteristics such as fluidity and packaging of the lipid hydrophobic tails. Impairment of membrane fluidity and lipid assembly may also modify the functions of membrane-associated proteins. As a consequence, numerous studies have shown that some biological/pharmacological effects, such as their antitumoral activity, can be attributed to the changes induced by flavonoids in some membrane-associated proteins [6,7,8,9,10].

**Figure 1:**
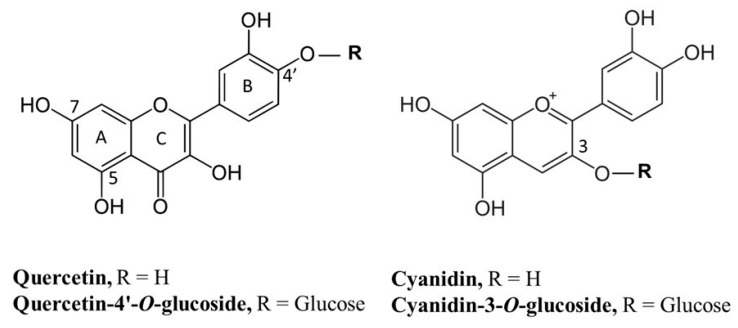
Chemical structures of the four flavonoids used in our study.

Quercetin (3,3′,4′,5,7-pentahydroxyflavone) (Figure 1) is the most representative dietary bioflavonoid, and is widely present in fruits and vegetables such as broccoli, cabbage, onions, apples, berries, cherries, red grapes, and tea.

It can occur as free aglycone, although most frequently it is present as a conjugated water-soluble glycosylated compound originating in the high number of quercetin derivatives found in plants. Quercetin and its derived glycosides have been found to possess different pharmacological activities including antiaging, antiviral, anti-cancer, and anti-inflammatory properties [11,12]. Cyanidin ((2-(3,4-dihydroxyphenyl) chromenylium-3,5,7-triol) (Figure 1) is an anthocyanidin cation, with a characteristic red-purple color, which is more often attached to a sugar moiety conjugated at the C3 hydroxyl group in the C-ring. The most common derivative is represented by cyanidin-3-*O*-glucoside (Figure 1). Cyanidin and its glycosides are particularly abundant in red fruits, apples, plums, red cabbage, and red onion. In addition to being responsible for the coloration of flowers, fruits, and vegetables, these specialized metabolites also possess many beneficial health effects, including antimicrobial, neuroprotective, antithrombotic, and anti-inflammatory activities [13,14]. Concentration of flavonoids may vary from one plant species to another or even between different plant organs. In one of our previous studies we showed that quercetin-4′-*O*-glucoside (Figure 1) and quercetin were the most abundant flavonoids of the hydroalcoholic extract from the dry outer scales of the bulb of *A*. *cepa* var. Tropea, “Cipolla Rossa di Tropea Calabria-PGI” (Tropea red onion). Cyanidin-3-*O*-glucoside was also present in this extract, likely contributing to the color of the bulb’s red scales. Results obtained from the biological assay demonstrated that the extract could significantly inhibit pancreatic lipase in vitro in a dose-related manner suggesting, its potentiality as an anti-obesity agent [15]. In addition, in the same study, it was shown that the hydroalcoholic extract of *A*. *cepa* var. Tropea was able to interact with lipids and permeabilize the planar lipid bilayer (PLM) through the formation of transient conductive units. The capacitive variations that have been observed following the formation of the conductive units indicated an increase in membrane thickness, which could be due to a stiffening and different packing of the phospholipid tails compared to what was observed in the PLM without the addition of the extract. The inhibition of pancreatic lipase activity shown in our study was interpreted as being mediated by changes in the membrane biophysical traits induced by the insertion of the extract components in the cellular membrane, in agreement with the results obtained from experiments with PLMs.

In the present investigation, we performed a systematic study on the interaction of four selected polyphenolic compounds (quercetin, quercetin-4′-*O*-glucoside, cyanidin, and cyanidin-3-*O*-glucoside) with PLM made of DOPS:DOPE:POPC in order to evaluate the following: their ability to integrate into PLM and form channel-like events; the effect of glycosylation on the ability to interact with PLM compared to that of aglycones; and their type of interaction with membrane lipids. The obtained results from this study may provide insights into the localization and modification of membrane fluidity by monitoring the electrical parameters of PLM in the presence of the four tested flavonoids, contributing to a better understanding of some of their biological activities and possibly those of plant extracts with a similar composition. To the best of our knowledge, data on the interaction of flavonoids with DOPS:DOPE:POPC PLMs, a surrogate of intestinal membranes [16], are reported here for the first time. Nevertheless, according to our best information, this is the first study on the interaction of cyanidin and cyanidin-3-*O*-glucoside with PLMs.

## 2. Materials and Methods

### 2.1. Chemicals

Dioleoyl-phosphatidylserine (DOPS), dioleoyl-phosphatidylethanolamine (DOPE), salts, and other basic chemicals used in PLM studies were obtained from Sigma (Munich, FRG, analytical grade); palmitoyl-oleoyl-phosphatidylcholine (POPC) was purchased from Avanti Polar Lipids, (Alabaster, AL, USA). Quercetin was bought from Extrasynthese, quercetin-4′-*O*-glucoside from Sigma-Aldrich, and cyanidin and cyanidin-3-*O*-glucoside were bought from Phytolab GmbH & Co.KG (Vestenbergsgreuth, Germany). All other chemicals were of high purity from commercial sources.

### 2.2. Electrophysiological Experiments

Channel-like activity was recorded in PLMs of DOPS:DOPE:POPC (27:27:18, w:w:w) in 1% n-decane. Bilayers were formed across a 300 μm hole in a Teflon partition separating two Teflon chambers (volume 4000 µL) that held symmetrical 1M KCl solutions, pH = 7, temperature 23 ± 1 °C. The aqueous solutions were used unbuffered. The salts used in the experiments were of analytical grade.

Stock solutions were prepared by dissolving the test samples in distilled water under stirring to a final concentration of 5 mg/mL. In four different experimental sets, 16 µL of stock solutions was added to the *cis* side of the PLM and stirred to have a final concentration of 0.02 mg/mL.

The membrane current was recorded on a chart recorder for further analysis. The two chambers, called *cis* and *trans*, were connected to the amplifier head stage by Ag/AgCl electrodes in series with a voltage source and a highly sensitive current amplifier (OPA 129). The single-channel instrumentation had a time resolution of 1–10 ms depending on the magnitude of the single-channel conductance. The polarity of the voltage was defined according to the side where the different compounds were added (the *cis* side). A *trans*-negative potential (indicated by a minus sign) means that a negative potential was applied to the *trans* side, the compartment opposite the one where the compounds were added. These experiments are considered to be a sensitive tool to study channel-forming substances [17,18].

The phenomenology of the substances’ incorporation and channel-like events formation was studied as follows:

- Conductance was determined by measuring by hand the amplitude of channel-like events. The single channel data, filtered at 300 Hz, were obtained from at least three experiments (more than 150 single channel-like events) performed on different days. For each experiment, a histogram of the conductance amplitude distribution for each experiment was constructed and fitted by a Gaussian distribution function. Results were expressed as central conductance ± standard error (*Λ_c_* ± SE) and analyzed by the ANOVA–Tukey test and the Student’s *t* test. A value of *p* < 0.05 was considered significant. The Gaussian distribution function, ANOVA test, Student’s *t* test, and the fitting procedures were performed using the GraphPad Prism 3 software (GraphPad Prism^TM^ version 3.0).
- To determine the PLM capacitance, we used a calibration curve obtained by simulating the membrane capacitance with a discrete set of capacitances of known values, *C_n_*, and by measuring the corresponding output voltage, *V_lh_*. Obtained data were fitted by the following formula:
  $$V_{lh}=\frac{A\times B}{\left(B+C_{n}\right)}$$
  in which *A* and *B* are free parameters deriving by the fitting procedures and their values used to transform the *V_lh_* value into capacitance data [19].
- To determine the frequency (number of channel-like events in 60 s), any detection of channel-like events was counted as successful. Results were expressed as frequency ± standard deviation (F ± SD).
- The following formula was used to calculate the size of the four flavonoids channel-like event:
  $$\Lambda_{c}=\frac{\left(\sigma \times \pi \times r^{2}\right)}{d}$$
  where *Λ_c_* is the central conductance, *σ* is the specific conductibility of the solution filling the channel, *r* is the channel-like event radius, and *d* is its length.

## 3. Results

### 3.1. Membrane Stability

First, we tested membrane stability by performing control experiments with the lipid mixture only to exclude non-specific effects. No change in the parameters of conductance (25 pS) and capacitance (0.30 µF/cm^2^) was observed for long periods of time (15 h) at the applied voltages of ±80 and ±100 mV or when the bilayer was broken and withdrawn by the operator.

In all experiments on DOPS:DOPE:POPC PLMs, before adding the tested polyphenolic compounds, the basic conductance (25 pS) and capacitance (0.30 µF/cm^2^) of the PLMs were monitored at the applied voltage of ±100 mV remaining constant for 12–15 min and no channel-like activity was observed (phase of membrane stabilization).

### 3.2. Behavior of the Four Flavonoids with DOPS:DOPE:POPC PLMs

To test the capability of the four flavonoids to incorporate into PLM and form channel-like events, four different sets of experiments were carried out in which the final concentration of the four compounds was 0.02 mg/mL. The addition of the four compounds to the *cis* side of the medium facing the membrane, regardless of the type of compound, was performed at the applied voltage of 80 mV (addition voltage).

After the membrane stabilization and the addition of the four compounds to the *cis* side of the medium facing the bilayer, we observed that the capacitance decreased slowly from the base value, reaching a minimum value in a time depending on the flavonoid used in the assay. After this time, the capacitance began to increase, reaching a lower value than that of the base capacitance which remained nearly constant until the end of the experiment. After reaching stable capacitance values, the interaction of the four flavonoids with the membrane appeared at an applied voltage of 80 mV as paroxystic activity that, in a few cases, led to membrane destabilization until rupture. In the latter case, the operator promptly withdrew the membrane. Paroxystic activity occurred for a period of time that depended on the compound used and then the interaction with the membrane gradually appeared as multilevel discrete non-random current fluctuations, compatible with channel-type openings and closures (multilevel channel-like activity). Current fluctuations showed different levels of conductance alternating with periods of quiescence and paroxystic activity. After the appearance of multilevel channel-like activity, the applied voltage was lowered as far as ±20 mV and the channel amplitude was monitored. Channel-like activity for each of the four flavonoids was recorded in the voltage range of ±20 to ±80 mV; each voltage was applied for 60 min starting from 80 mV (i.e., 60 min at 80 mV, followed by 60 min at −80 mV, etc.).

For each applied voltage, experimental data of conductance levels were employed to construct a histogram of the conductance amplitude distribution showing that, at all applied voltages, there was one main conductance value. Histograms created at different applied voltages were fitted by a Gaussian distribution function and gave the central value of conductive unit conductance (*Λ_c_*) formed from each of the four flavonoids.

### 3.3. Insertion of Quercetin and Its Glucosylated Derivative into DOPS:DOPE:POPC PLM and Channel-like Activity

In the first and second experimental sets, we tested the ability of quercetin and quercetin-4′-*O*-glucoside to incorporate and form conductive units in DOPS:DOPE:POPC PLMs, respectively.

In the first experimental set, we added 16 µL of quercetin stock solution to the medium of the *cis* chamber and we monitored its interaction with the PLM. The capacitance value just before adding the substance was 0.32 ± 0.01 µF/cm^2^. After the flavonoid addition, the capacitance decreased slowly and reached the value of 0.11 ± 0.02 µF/cm^2^ over a period of about 60 min. After this time the capacitance increased, reaching the value of 0.28 ± 0.03 µF/cm^2^, which remained almost constant until the end of the experiment (Table 1).

**Figure 2:**
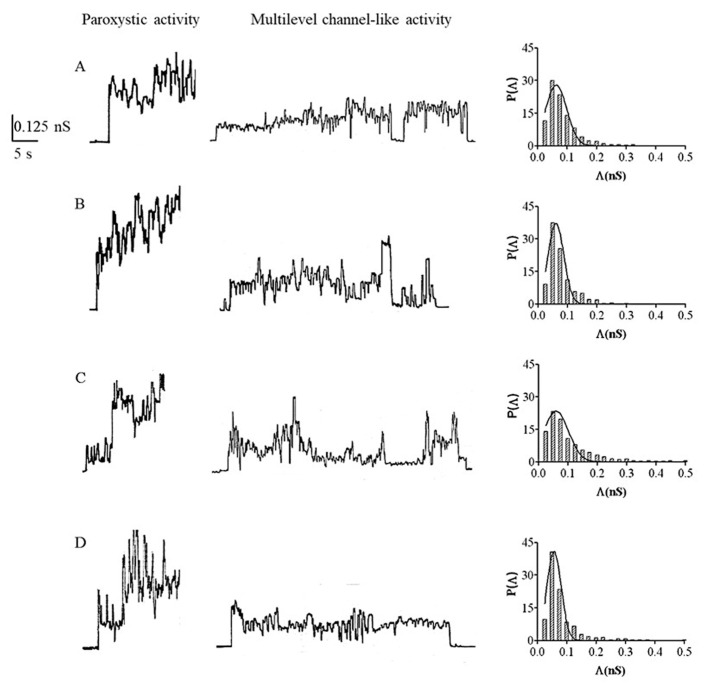
Quercetin, cyanidin, and their *O*-glucosides’ channel-like activity in DOPS:DOPE:POPC PLMs. Example of chart recordings of channel-like activity of quercetin (**A**), quercetin-4′-*O*-glucoside (**B**), cyanidin (**C**), and cyanidin-3-*O*-glucoside (**D**) in PLMs made up of DOPS:DOPE:POPC with associated histograms of the conductance fluctuations. The histograms of the probability, P(Λ), for the frequency of a given conductivity unit were fitted by a Gaussian distribution, which is shown as a solid curve. Experiments were performed in the presence of the different compounds (0.02 mg/mL) added to the *cis* side, while the aqueous phase contained 1 M KCl (pH 7) and T = 23 ± 1 °C. Applied voltage was set to 40 mV.

About 15 min after reaching the constant value of capacitance, the interaction of quercetin with the bilayer appeared at the applied voltage of 80 mV as paroxystic activity which remained constant for 30–35 min. Paroxystic activity gradually disappeared, while a multilevel channel-like activity appeared. It is important to observe that the interaction of quercetin with PLMs was characterized by alternating periods of multilevel channel-like activity, periods of quiescence, and periods of paroxystic activity at all the applied voltages. Figure 2 is an example of the chart recordings of quercetin channel activity.

**Figure 3:**
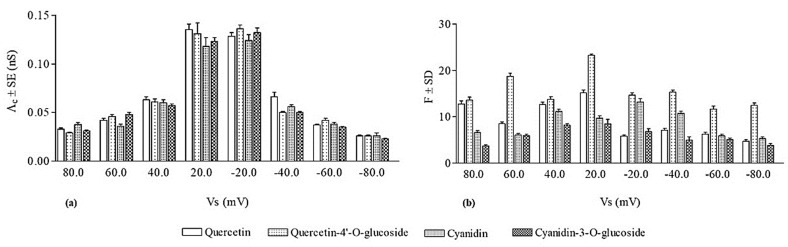
Biophysical and statistic parameters of four flavonoids’ channel-like events in DOPS:DOPE:POPC PLM. (**a**) The mean conductance (*Λ_c_* ± SE) and (**b**) frequency (F ± SD) of four flavonoids’ channel-like events at different applied voltages. The minimum and maximum number of channel-like events considered (N) out of a total number of channel-like events considered (Nt) was: Quercetin = 167 < N < 772, Nt = 3599; Quercetin-4′-*O*-glucoside = 155 < N < 786; Nt = 3187; Cyanidin = 228 < N < 616, Nt = 2895; Cyanidin-3-*O*-glucoside = 122 < N < 528, Nt = 1718.

As mentioned above, we calculated the central value of the channel-like events’ conductance (*Λ_c_*) formed from quercetin at all the applied voltages. Figure 3 shows the values of *Λ_c_* ± SE obtained in this experimental set.

The occurrence frequency of quercetin channel-like events was calculated and the obtained values (F ± SD) are reported in Figure 3. It is interesting to note that the frequency of channel-like events was significantly higher at positive applied voltages than at the negative ones.

Another biophysical parameter that characterizes a conductive unit is its duration. The duration of quercetin channel-like events was within a range from 0.75 to 2.25 s.

In the second experimental set, we monitored the interaction of quercetin-4′-*O*-glucoside with the membrane. When the glucosylated compound was added to the *cis* side of the medium facing the PLM, the capacitance value was 0.32 ± 0.03 µF/cm^2^. After its addition, the capacitance decreased, reaching the minimum value of 0.12 ± 0.02 µF/cm^2^ in a period of time of 30 min, after which the capacitance increased until it reached values of 0.26 ± 0.01 µF/cm^2^, which remained constant until the end of experiment (Table 1). About 30–35 min after reaching the capacitance constant value, the interaction of quercetin-4′-*O*-glucoside with PLM appeared at the voltage of 80 mV as paroxystic activity. The paroxystic activity lasted about 20 min; then, the multilevel channel-like activity gradually appeared. The quercetin-4′-*O*-glucoside interaction was characterized by periods of channel-like activity that lasted longer than periods of quiescence and paroxystic activity at all the applied voltages. Examples of chart recordings of quercetin-4′-*O*-glucoside channel activity are shown in Figure 2.

As for quercetin, and for its glucosylated derivative, the central conductance values (*Λ_c_* ± SE) and the frequency values (F ± SD) were calculated (Figure 3). It is worthy of note that the values of frequency obtained at positive voltages were not significantly different from those obtained at negative applied voltages.

The duration of the glucosylated quercetin channel-like events was in the range of 1.25 to 4.25 s.

Comparing the biophysical parameters (*Λ_c_*, F, and duration of channel-like event) obtained for quercetin and for its glucosylated derivative, it is noteworthy that the *Λ_c_* values are not significantly different at all the applied voltages; the frequency values of glucosylated quercetin tend to be higher at positive applied voltages and significantly higher at negative applied voltages than those of quercetin; and the duration of the channel-like events occurring in glucosylated quercetin is greater than that obtained for quercetin.

### 3.4. Insertion of Cyanidin and Its Glucosylated Derivative into DOPS:DOPE:POPC PLM and Channel-like Activity

In the third and fourth experimental sets, we tested the ability of cyanidin and cyanidin-3-*O*-glucoside to incorporate and form conductive units in DOPS:DOPE:POPC PLMs, respectively.

After the addition of cyanidin to the *cis* side of the medium facing the membrane, we observed that the capacitance decreased slowly from the basic value of 0.30 ± 0.01 µF/cm^2^, reaching the minimum value of 0.12 ± 0.01 µF/cm^2^ in a time of about 45–50 min. After this time the capacitance increased, reaching a value of 0.26 ± 0.02 µF/cm^2^ (Table 1). About 10 min after reaching stable capacitance values (0.26 ± 0.02 µF/cm^2^), the interaction of cyanidin with PLM was evident at the applied voltage of 80 mV as paroxystic activity that occurred for about 25–30 min, and then the cyanidin interaction gradually developed into a multilevel channel-like activity. It is interesting to note that cyanidin channel-like activity alternated with periods of quiescence and brief periods of paroxystic activity. Examples of chart recordings of cyanidin channel activity are shown in Figure 2.

For each applied voltage, we constructed the histogram of the conductance amplitude distribution and, using a Gaussian distribution function, we calculated the central value of cyanidin conductive unit conductance (*Λ_c_*). *Λ_c_* values (*Λ_c_* ± SE) as a function of the applied voltage are reported in Figure 3.

The frequency of cyanidin channel-like events was determined for each applied voltage. Figure 3 shows that the frequency values are significantly higher in the applied voltage range ±20–±40 mV than those observed at applied voltages of ±80 and ±100 mV. This result could be due to the fact that the periods of paroxystic activity are more frequent at higher applied voltages, during which the number of channel-like events could not be rigorously analyzed.

Another parameter that was calculated is the duration of the observed channel-like events, which was within the range from 0.75 to 1.25 s.

In the fourth experimental set, 16 µL of cyanidine-3-*O*-glucoside stock solution (5 mg/mL) was added to the medium of the *cis* chamber when the values of basic conductance and capacitance were stable (25 pS and 0.31 µF/cm^2^, respectively). Similar to what was observed for cyanidin, in this experimental set the capacitance also first slowly decreased from the basic value, reaching the minimum value of 0.10 ± 0.03 µF/cm^2^ in a time of 35 min, and then increased, reaching capacitance values of 0.25 ± 0.03 µF/cm^2^ (Table 1). About 25–30 min after reaching stable capacitance values (0.25 ± 0.03 µF/cm^2^), the interaction of cyanidin-3-*O*-glucoside with PLM appeared as paroxystic activity that lasted about 45 min. In most of the experiments, paroxystic activity led to membrane destabilization until rupture. After PLM breakage and withdrawal, the paroxysmal activity occurred promptly and persisted for about 10–15 min, after which the activity gradually showed a multilevel channel-like activity at the applied voltage of 80 mV. Channel-like activity alternated with periods of quiescence and periods of paroxysmal activity at all applied voltages used in this investigation. It should be highlighted that in this fourth experimental set the periods of quiescence were longer and the periods of paroxystic activity more frequent than those observed in the third experimental set. Examples of chart recordings of cyanidin-3-*O*-glucoside channel activity are shown in Figure 2.

Figure 3 reports the central conductance values (*Λ_c_* ± SE) obtained by fitting the experimental data with a Gaussian function and frequency values (F ± SD) of the cyanidin-3-*O*-glucoside.

As for cyanidin, we also determined the lifetime of channel-like events induced by its glucosylated derivative. The duration of channel-like events was within the range from 0.25 to 0.75 s.

The results obtained from two experimental sets indicated that cyanidin and its glucosylated form are able to penetrate into the hydrophobic domain of PLMs and cause channel-like events preceded by the paroxystic activity, although the periods of paroxysm were more frequent for the glucosylated derivative. Comparing the biophysical parameters of the conductive unit formed by cyanidin and its glucosylated derivative (Figure 3), it is possible to observe at all the applied voltages that the *Λ_c_* ± SE values obtained for the two compounds were not significantly different, and the F ± SD values calculated for cyanidin were higher than those for its glucosylated derivative.

The lifetime of the conductive unit formed by cyanidin-3-*O*-glucoside was shorter than that of cyanidin.

## 4. Discussion

In recent decades, functional foods have received increasing attention from both economic and scientific points of view. By definition, functional foods are a category of food which, in addition to their basic nutritional properties, display a positive effect on one or more physiological functions, helping to ameliorate or preserve health conditions. The possibility of improving health and physical well-being has prompted many food industries to invest in the functional food sector. The health-positive effects of functional foods can be attributed to the presence of bioactive metabolites responsible for their beneficial effects. This explains the interest in research to identify these molecules or mixtures of molecules and understand the mechanisms underlying their biological effects.

Flavonoids are phytochemicals produced in numerous vegetables and fruits, the consumption of which has been documented to display a positive influence on human health. As an example, flavonoid intake is inversely related to heart disease mortality and to the frequency of myocardial infarction [20]. Moreover, flavonoids play a protective role for the vascular system by carrying oxygen and nutrients to the cells [21,22]. The numerous positive effects of flavonoids on human health are now recognized and many studies have been undertaken to clarify the mechanisms of action by which these plant products exert their protective effects, as reported in the recent comprehensive review by Abou Baker DH [5].

The mechanisms of flavonoids’ beneficial actions involve enzymes and receptors, both intracellular and associated with the cell membrane system, as well as with the plasma membrane itself. Thus, the lipid bilayer represents an important target of flavonoids, whose interaction is modulated both by the type of lipids that make up the membranes and by the structural type of flavonoid involved in the interaction. As a result of this interaction, some biophysical parameters of the membranes, such as fluidity and thickness, can be modified, thus contributing to the biological activity and therapeutic potentials of these compounds. Numerous studies indicated that the lipid composition of liposomes influences the ability of flavonoids to cause their aggregation. Thus, liposomes containing phosphatidylserine and phosphatidylinositol were shown to aggregate more easily than those containing cholesterol in the presence of highly hydrophobic flavonoids, suggesting that those structural types can enter deeper into the lipid bilayer. In contrast, less hydrophobic flavonoids show a higher affinity for the polar aqueous interface and tend to localize on the surface of the phospholipid liposomes, promoting their aggregation through a decrease in the membrane hydration [23]. The interplay between lipids and flavonoids has been the subject of numerous studies [10,23,24,25,26,27,28,29,30].

In our previous paper [15], we studied the interaction of the hydroalcoholic extract from the dry outer scales of the Tropea red onion with PLMs made up of POPC:DOPE:DOPS whose composition is similar to that of intestinal membranes. In the present study, we monitored the interactions between some of the flavonoids identified in the above extract, that is, quercetin and its glucosylated derivative quercetin-4′-*O*-glucoside, cyanidin-3-*O*-glucoside, and cyanidin, with PLMs having the same lipid composition as that used in our previous study. Our aim was to support the results obtained with the whole raw extract and to try to clarify the role played by its main components in the mechanism of action underlying the interaction with PLMs and some of their biological activities.

The electrophysiological method of PLMs is a useful tool for obtaining information on the molecular mechanism of chemicals that interact with membrane lipids. Many substances (proteins, peptides, or drugs) that are active at the membrane interface promote ionic current flow across the membrane (i.e., increase membrane conductance) when they incorporate into the lipid bilayer and form conductive units [31,32,33,34,35,36,37]. Several pore models formed by different interface active compounds have been proposed and the different characteristics of the current signals have been associated with the different models, as reported by Bertrand et al. [38].

Results obtained in our study indicate that the four tested flavonoids are able to penetrate into the hydrophobic core of PLMs and form channel-like events, which show common characteristics of interaction with bilayer lipids, as shown in Figure 2.

The first behavior common to the four compounds was the variation in capacitance which, after the compounds’ addition to the medium (*cis* side) facing the PLM, decreases, reaching a minimum value, before subsequently increasing until it reaches a stable value, which is always lower than the basic value of the capacitance. The decrease in the capacitance from the basic value to the minimum value could be due to the adsorption of the flavonoids on the PLM surface until the achievement of a lipid/chemical ratio critical for the insertion of the compounds into the hydrophobic core of the bilayer, as shown for other products active at the lipid interface [32,37] and also observed by us for the total hydroalcoholic extract from the bulbs’ outer scales of onion var. Tropea [15]. It is important to note that the times in which adsorption took place and the achievement of the critical lipid/chemical ratio (longer times for the aglycones than for their glucosylated derivatives) depended on the tested compounds (Table 1) and were modulated by their lipophilicity. It is plausible to hypothesize that quercetin and cyanidin, which are more lipophilic than their related glucosylated derivatives, localize below the polar heads, i.e., on the surface of the hydrophobic core, thus taking a longer time to reach this localization site than their glucosylated derivatives which localize at the level of the polar heads of the bilayer. This result is in line with that found by other authors who used liposomes as a lipid model. Eid and colleagues showed that quercetin interfaces between the hydrophobic core and polar head groups of the membrane lipids with the larger aromatic ring roughly parallel to the *z*-axis of the bilayer compared to the smaller phenyl ring [39], while its glycosides appear to preferentially locate at the polar head groups. Sanver and co-workers conducted a study on the bilayer structural changes induced by quercetin. The results of this study showed that quercetin is localized under the hydrophilic head region at 12.2 ± 2.2 Å from the membrane center causing membrane thinning [10]. Cyboran-Mikolajczyk and colleagues studied the interplay of cyanidin and some of its 3-*O*-glycosylated derivatives with a liposome membrane made up of POPC [40]. The results obtained with fluorescence spectroscopy indicated that cyanidin and its 3-*O*-glycosides cause changes in the hydrophobic domain of the membrane in the following order: cyanidin > cyanidin with a monosaccharide moiety > cyanidin containing a disaccharide moiety. The order parameter (S) and the wobbling diffusion coefficient (Dw) of the DHP probe, obtained from the analysis of the anisotropy decay data of the probe, are significantly influenced by cyanidin and less significantly by its glycoside derivatives. The S parameter, which approximates the order of the lipid acyl chain, increases, while the Dw parameter, which is correlated with the lipid dynamics, decreases, in the presence of cyanidin and its derivatives. These results indicate that cyanidin affects deeper regions of the bilayer than its glycosides by localizing at the hydrophobic–hydrophilic interface, while its derivatives localize near the lipid polar heads. The effect of all these compounds on the two parameters, S and Dw, are also indicative of a decreased membrane fluidity [40]. The results obtained by us for cyanidin and its 3-*O*-glucoside with the electrophysiological measurement of the biophysical parameters of PLMs are in good agreement with those reported by Cyboran-Mikolajczyk S et al. [40]. To the best of our knowledge, no studies of PLMs have been carried out before for cyanidin and its glycosides.

The increase in the capacitance from a minimum value to a stable value recorded during our experiments could be due to the change in orientation of the tested molecules that, after being adsorbed, penetrate the hydrophobic core of the membrane, inducing a membrane thinning (Table 1). In accordance with their lipophilicity, the time that elapses between the achievement of the constant value of capacitance and the manifestation of paroxystic activity is shorter for quercetin and cyanidin than for their glucosylated derivatives. Our results are in agreement with those found by other authors for quercetin. Movileanu et al. interpreted changes in transmembrane capacitance and conductance measured in the presence of quercetin as being due to the capacity of the flavonoid to intercalate into the membranes of phosphatidylcholine between the flexible acyl chains of the phospholipids [41]. Moreover, Kruszewski et al. investigated the impact of quercetin on the electrical properties of model lipid bilayers and human glioblastoma cells (cellular lines LN-229 and LN-18) [42]. The results obtained with model lipid membranes showed that at alkaline pH quercetin localizes close to the polar heads, changing the structure and properties of the membrane, while at acid pH it penetrates the hydrophobic region [42]. It is also interesting to note that the location of flavonoids depends on the saturation of the fatty acids of the phospholipids. The flavonoids are localized either in the region of the polar heads of the lipid bilayer, when it is made of saturated phospholipids such as DPPC [24]; or in the hydrophobic domain of the bilayer, when it is made of unsaturated phospholipids such as POPC [8], SLPC [43], POPS, and POPE [44]. Model lipid systems containing unsaturated phospholipids are more fluid than those containing saturated lipids [44].

The second behavior common to the four tested flavonoids was represented by the way they interacted with the lipid bilayer. After reaching a stable value of capacitance, the interaction appeared first as paroxystic activity and then as multilevel channel-like activity. The paroxystic activity can be attributed to the insertion of the molecules into the hydrophobic core of the membrane, thereby causing “defects” in the lipid bilayer. The paroxystic phase, which had almost the same duration for all the four flavonoids tested in this study, gradually subsided, while the multilevel channel-like activity appeared, indicating that the tested molecules assembled to form stable conductive units. The characteristics of the current signals observed in the experimental chart recordings indicate that the configuration of the conductive unit formed by the four flavonoids could be toroidal [38]. In the toroidal configuration, the pore lumen is formed both by the tested molecules inducing pore formation and by the polar heads of the phospholipids. This configuration is in agreement with the amphipathic structure of flavonoids which have polar substituents linked to a non-polar backbone. A similar result was also obtained in our previous study on the interaction of resveratrol with PLM of different lipid composition [37].

It is notable that the *Λ_c_* values, at all applied voltages were not significantly different for the four flavonoids (Figure 3), indicating that the number of molecules that assemble in the lipid bilayer to form the conductive unit is the same for all the compounds. Indeed, the conductive unit size, depending on the molecule assembly, can be obtained from the conductance values, assuming that the conductive unit is a water-filled hole. In this case, in the DOPS:DOPE:POPC membrane (with an average thickness of 5 nm) and at an applied voltage of 20 mV, the conductive unit diameter of quercetin/quercetin-4′-*O*-glucoside and cyanidin/cyanidin-3-*O*-glucoside was 2.8/2.7 nm and 2.6/2.6 nm, respectively. The conductance is a biophysical parameter related to the ions’ current through the bilayer when an active compound at the interface penetrates the hydrophobic core of the bilayer forming conductive units. Our results indicate that the four flavonoids, regardless of their glucosylation, are able to influence the PLM capacitance and to form conductive units by inserting themselves into the bilayer (consisting of unsaturated phospholipids), probably in close proximity to the double bonds of fatty acids protecting the phospholipids from oxidation reactions. This information, although indirect, could explain the anti-peroxidation effect displayed by the four flavonoids which, acting as electron donors to free radicals, neutralize and inhibit their ability to damage the cell membrane, as found for other polyphenols [37,45,46]. Furthermore, the antioxidant effects of flavonoids may derive not only from their capacity to interact with reactive oxygen species, but also from their ability to modify the fluidity of the bilayer. The stiffening of the lipid bilayer, induced by the incorporation of the four flavonoids, decreases the lateral diffusion of oxidizable lipids by reducing the kinetics of oxidative reactions [43].

The duration in the open state of a channel-like event is a parameter correlated with its stability. The duration values obtained for quercetin/quercetin-4′-*O*-glucoside are in the range of 0.75–2.25/1.25–4.25 s, respectively, indicating that the conductive unit formed by the glucosylated derivative is more stable than that formed by the aglycone. In contrast, the duration of the channel-like events for cyanidin/cyanidin-3-*O*-glucoside is in the range of 0.75–1.25/0.25–0.75 s, respectively, indicating that, for these two compounds, the conductive unit formed by the aglycone is more stable than that of its glucosylated derivative. Membrane interactivity of flavonoids depends on some structural requisites such as the presence of a hydroxyl group at C3 of the heterocyclic C ring, which makes flavonols, such as quercetin, more reactive than their structurally related flavones [44]. Affinity to membrane lipids and potency to rigidify them also increases with the -5,7-dihydroxylation of ring A (as in quercetin and cyanidin). Another significant determinant for flavonoids’ interaction with the lipid bilayer is the number of OH groups in the B ring (the lower the number, the higher the affinity to the bilayer) [44]. Thus, the two aglycones used in our study, quercetin and cyanidin, possess all the structural requisites to be able to interact efficiently with the used PLMs. Generally, glycosylation makes flavonoids less lipophilic and larger in size, therefore reducing their affinity for membrane lipids. Based on these structural requirements, the above results can be explained by the different substitution at C3 on ring B; that is, the glucose moiety at C3 of cyanidin-3-*O*-glucoside makes this compound less reactive than the cyanidin aglycone. Nevertheless, we can suggest that, compared to cyanidin/cyanidin-3-*O*-glucoside, glucose substitution in C4′ in quercetin-4′-*O*-glucoside does not greatly affect its lipid affinity. Moreover, compared with its aglycone (and with cyanidin-3-*O*-glucoside), quercetin-4′-*O*-glucoside has fewer free OH groups in ring C; that is, a higher membrane interactivity is expected for this compound, which could also explain its higher stability.

The frequency values of the quercetin-4′-*O*-glucoside channel-like event tend to be higher, especially at negative voltages, than those of quercetin, indicating a greater turnover of channel-like events. Nevertheless, the frequency values of the channel-like event observed for cyanidin tend to be higher than those of its glucosylated derivative. These results are in line with those of the conductive unit lifetime, reinforcing the hypothesis that the glucosylated hydroxyl groups’ location affects the affinity to the lipid bilayer and the stability of its assembled conductive units. It is noteworthy that the lifetime and frequency of the conductive unit formed by quercetin and quercetin-4′-*O*-glucoside are higher than those observed for cyanidin and its glucosidic derivative.

## 5. Conclusions

**Figure 4:**
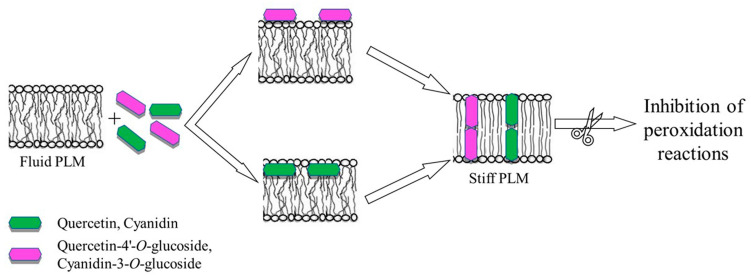
Schematic model of the four flavonoids’ interaction with DOPS:DOPE:POPC PLM. Quercetin-4′-*O*-glucoside and cyanidin-3-*O*-glucoside adsorb onto the membrane surface (top), while quercetin and cyanidin localize at the interface between the polar region of head groups and the hydrophobic core of the bilayer (bottom). The incorporation of the four flavonoids into the membrane and the formation of conductive units influences the electrical properties of PLM.

The results of this study show the following: the four analyzed flavonoids are able to interact with the PLM made of DOPS:DOPE:POPC and to form channel-like events through a first step in which the molecules are adsorbed on the membrane surface and a second step in which they assemble into the membrane to form stable conductive units; in accordance with the values of their adsorption time, quercetin and cyanidin are located at the interface between the hydrophilic heads and the non-polar core of the bilayer, while their glucosylated derivatives are localized more superficially; and the membrane capacitance is influenced by the interaction of the four flavonoids with the lipids of the bilayer (Figure 4).

These results provide indications about the localization of the four flavonoids in the lipid bilayer made up of DOPS:DOPE:POPC and about the changes in the biophysical parameters of the membrane, such as fluidity and thickness, helping to clarify some mechanisms of action underlying some of the biological activities of polyphenolic compounds.

To the best of our knowledge, the interaction of quercetin, quercetin-4′-*O*-glucoside, cyanidin, and cyanidin-3-*O*-glucoside with DOPS:DOPE:POPC PLMs, a surrogate of the intestinal membrane, has never been previously monitored.

These results reinforce the concept, widely expressed in the literature, that cell membranes are one of the main targets of flavonoids. These phytochemicals can cross the plasma membrane to reach an intracellular target or can influence its biophysical parameters, thus regulating some of their biological effects such as membrane protein modulation, and anti-inflammatory, anti-cancer, and antimicrobial effects. Finally, these results fully support the inhibitory effect of the total extract from the dry outer scales of the bulbs of *A. cepa* var. Tropea on pancreatic lipase, as found in our previous study [15]. Evidence obtained here also suggests that quercetin and its glucosyl derivative, representing the most abundant constituents of the above extract, are likely to be the main constituents responsible for the interaction with the PLMs. The interaction modality (paroxysmal activity and multilevel channel-like activity) and the capacitive trend in the presence of the four tested flavonoids presenting characteristics common to those of the extract, support this concept.

## Figures and Tables

**Table 1:** Capacitance variation in DOPS:DOPE:POPC PLM. Mean values of the membrane capacitance calculated as follows: just after the addition of each tested flavonoid (Ci ± SE); when the capacitance reached a minimum value (Cm ± SE); and when the capacitance reached a stable value (Cs ± SE). The time between the capacitance value immediately after adding the four flavonoids (Ci) and the minimum capacitance value (Cm) is shown in the fifth column (Time). The mean value was obtained from at least four experiments.

Compound	Ci ± SE µF/cm^2^	Cm ± SE µF/cm^2^	Cs ± SE µF/cm^2^	Time s
Quercetin	0.32 ± 0.01	0.11 ± 0.02	0.28 ± 0.03	60
Quercetin-4′-*O*-glucoside	0.32 ± 0.03	0.12 ± 0.02	0.26 ± 0.01	30
Cyanidin	0.30 ± 0.01	0.12 ± 0.01	0.26 ± 0.02	50
Cyanidin-3-*O*-glucoside	0.31 ± 0.02	0.10 ± 0.03	0.25 ± 0.03	35

## Data Availability

Not applicable.
